# Native seed, soil and atmosphere respond to boreal forest topsoil (LFH) storage

**DOI:** 10.1371/journal.pone.0220367

**Published:** 2019-09-16

**Authors:** Dean D. Mackenzie, M. Anne Naeth

**Affiliations:** 1 Vertex Resource Group Ltd, Sherwood Park, Alberta, Canada; 2 Department of Renewable Resources, University of Alberta, Edmonton, Alberta, Canada; Chinese Academy of Forestry, CHINA

## Abstract

During mining topsoil is salvaged and stockpiled until ready for reclamation, stockpiling can have detrimental effects on seed viability and soil quality. Research has assessed effects of salvage and placement depth of forest topsoil on plant community establishment, with little work on effects of storage, particularly in the boreal forest. Our research assessed boreal forest topsoil storage methods to determine effects on soil chemical and physical properties, native seed viability and germination and rhizome viability and emergence. Factors were topsoil stockpiling length, stockpile size, season of construction and soil texture. Four replicates of large and small stockpiles were constructed in the mineable oil sands, in northeastern Alberta. During construction seeds and rhizomes from a variety of native boreal plant species were buried within large (0.05, 1.0, 2.0, 4.0, 6.0 m) and small (0.05, 1.0, 3.0 m) stockpiles. Soil gas probes were installed at similar depths as seed and rhizomes were placed. Seeds and rhizomes were extracted eight months and sixteen months after construction; during that time soil samples were collected for various chemical analyses. Irrespective of stockpile size, the majority of species seeds and rhizomes buried below 1 m lost viability and did not germinate after eight months. Anaerobic soil conditions developed soon after construction and persisted at depths below 1.0 m in large stockpiles, and over time anaerobic conditions developed in smaller stockpiles. Only seeds of *Geranium bicknellii* and *Dracocephalum parviflorum* had a high survival rate in stockpiles; both species have hard seed coats and are physically dormant. Various soil nutrients increased in concentrations in their soluble forms after stockpiling. Direct placement of topsoil is a preferred soil handling technique; however, if topsoil has to be stockpiled increasing the surface area of stockpiles will help preserve some seed and rhizome viability.

## Introduction

In the energy sector, disturbances from exploration, development and operation usually require reclamation, which often means replacing salvaged topsoil. In many jurisdictions these disturbances in boreal forest must be reclaimed to diverse, self-sustaining plant communities similar to the surrounding region. Research on boreal forest topsoil (LFH, LFH mixed with upper mineral horizons) has provided industry with a means to use a local diverse seed source and fertile surface soil to meet reclamation requirements [1, 2]. Organic soil horizons (LFH), develop primarily from accumulation of leaves, twigs and woody materials with or without a minor component of mosses, and are normally associated with upland forest soils with imperfect drainage or drier [1,2]. Within this paper LFH refers to organic material from an upland forest and describes the mix of LFH layer and upper mineral soil, which can include the Ahe, Ae, upper B horizon or combinations of these horizons, over stripped during salvage and placed for reclamation in the oil sands region. Salvaging, storing and replacing LFH is now considered a best practice in Alberta [3]. Research has assessed effects of salvage and placement depth of forest topsoil on plant community establishment, with little work on effects of storage, particularly in the boreal forest.

Impacts of topsoil storage are recognized [4], with much of the work to date conducted in Australia. Soil quality changes include reduced organic matter, nitrogen, microbial activity, mycorrhizal density, earthworm populations and nutrient cycling [5, 6]. Losses of organic carbon occurred quickly in the upper 15 cm (28%) and 100 to 150 cm layer (12.6%) of Canadian stockpiled, prairie topsoil [7]. The magnitude of soil quality reduction is affected by texture and depth in the stockpile. Organic matter losses were greater in sandy (85%) than clay soil (32%) [8]. Total nitrogen decreased 100 mg kg^-1^ on stockpile surfaces and deeper in the stockpile after thirty months [9]. Anaerobic conditions were detected deep in stockpiles [10]. Soil gases, such as carbon dioxide and ethylene, increased with depth and clay content, with methane and nitrogen high even at shallow depths [8]. Dramatic increases in extractable manganese, ferrous iron and ammonium nitrogen occurred with increasing depth, with greater changes in clay than sandy soil. For example, in a clay texture stockpile, ferrous iron increased from 72 ug g^-1^ at 40 to 50 cm to 18,121 ug g^-1^ at 100 to 150 cm and in a sand texture stockpile from 371 ug g^-1^ to 4,800 ug g^-1^ at 100 to 150 cm [8]. In Alberta Canada, temperature in stored peat stockpiles fluctuated between 0 and 20°C throughout one year in the upper 50 cm, remaining relatively constant at below 10°C at greater depths [11]. In Australia small soil temperature differences were found between wet and dry stockpiles, with temperatures fluctuating (6 to 37°C) with seasonal changes in the upper 100 cm, but remaining constant (21 to 28°C) below [9].

Seed viability in stockpiles decreases with storage time and can occur quickly. A significant decrease in viability of 12 *Banksia* woodland species in Australia occurred after one month of storage; depth and storage time had little effect [12]. Another Australian study showed seed bank viability decreased 3 to 13% in 10 to 13 months [13]. After four months and four years propagule viability decreased significantly for most species in older piles at all depths in Derbyshire [14]. Establishment decreased considerably when using stockpiles as an inoculum on reclaimed Australian sites [15]. Various factors affect seed longevity in stockpiles including in situ germination, microbial pathogens and senescence [16]; seeds and rhizomes may be physically damaged during stockpiling.

Stockpiling effects could be different in cold environments such as boreal forest, than in warm climates. Rhizomes are a major contributor to secondary succession in the boreal [17], although no attempts have been made to assess rhizome viability in stockpiles. Effects of stockpile size on viability of propagules and soil quality has not been studied; our research aimed to answer the question what effects does storing boreal forest soil have on soil chemical, physical and biological properties? Our research assessed boreal forest topsoil storage methods to determine effects on soil chemical and physical properties, native seed viability and germination and rhizome viability and emergence. Factors were topsoil stockpiling length, stockpile size, season of construction and soil texture.

## Materials and methods

### Research site descriptions

Stockpiles were constructed in three areas near active oil sands mines in the central mixed wood subregion of the boreal natural region. Climate was cool temperate with short, cool summers and long, cold winters [18]. Mean annual temperature was 0.3°C. The 1944 to 2007 long term average annual precipitation was 471.2 mm, with 322.7 mm of rain and 148.5 cm of snow. Soils in the area are orthic gray luvisols and eluviated and orthic eutric brunisols [19]. Pre-disturbance vegetation was representative of the mixed wood boreal forest for each soil. Undisturbed orthic gray luvisols typically support *Picea glauca* (Monech) Voss (white spruce) and *Populus tremuloides* Michx. (trembling aspen) forests and orthic eutric brunisols support *Pinus banksiana* Lamb. (jack pine) and *Populus tremuloides* Michx.

### Seed and rhizome collection and processing

Seeds and rhizomes of 27 available native boreal plant species, representative of common families, life forms, dormancy mechanisms and seed sizes from unmined areas around the mines were hand collected between July and September. Species nomenclature followed Moss [20]. Forbs were *Anemone multifida* Poir. (cut leaved anemone), *Anemone patens* L. (prairie crocus), *Aralia nudicaulis* L. (wild sarsaparilla), *Cornus canadensis* L. (bunchberry), *Dracocephalum parviflorum* Nutt. (American dragonhead), *Fragaria virginiana* Duchesne (wild strawberry), *Geranium bicknellii* Britt. (northern cranesbill), *Maianthemum canadense* Desf. (lily of the valley), *Potentilla tridentata* Ait. (three toothed cinquefoil), *Rubus pubescens* Raf. (dewberry) and *Vicia americana* Muhl. (American vetch). Grasses and sedges were *Agropyron trachycaulum* var. trachycaulum (Cassidy) Malte (slender wheat grass), *Bromus ciliatus* L. (fringed brome), *Elymus innovatus* Beal. (hairy wild rye), *Oryzopsis pungens* (Torr.) A.S. Hitchc (jack pine rice grass) and *Carex aenea* Fern. (bronze sedge). Shrubs and trees were *Alnus crispa* (Ait.) Pursh (green alder), *Arctostaphylos uva-ursi* (L.) Spreng. (kinnickinick), *Prunus pensylvanica* (L.f) (pin cherry), *Ribes hudsonianum* Richards. (wild black currant), *Rosa acicularis* Lindl. (prickly rose), *Rubus idaeus* L. (wild red raspberry), *Shepherdia canadensis* (L.) Nutt. (Canada buffalo berry), *Vaccinium myrtilloides* Michx (blueberry), *Vaccinium vitis-idea* (L.) Nutt. (bog cranberry), *Viburnum edule* (Michx.) Raf. (low bush cranberry) and *Pinus banksiana* Lamb. (jack pine). Rhizomes from *Maianthemum canadense*, *Vaccinium myrtilloides* and *Arctostaphylos uva-ursi* were hand dug. Seeds were air dried at room temperature for two weeks before placement. Berries were macerated with a blender, screened and cleaned with tap water to prevent viability loss from fungi, air dried for two weeks, then stored in sealed jars in the dark at room temperature. Rhizomes were harvested one week prior to placement and stored at 4°C in a refrigerator.

Four replicates of 25 seeds from each species were used to study viability and germination prior to placement in stockpiles and the same number following stockpiling. Prior to placement in stockpiles, seeds of each species were placed in individual 5 x 8 cm permeable, black, mesh, sewn bags of 100% nylon, allowing them to be in ambient hydrologic and temperature conditions in the stockpiles yet facilitating their recovery from the stockpile. One quarter to one teaspoon (based on seed size) of heat sterilized sand was added to each bag to evenly distribute seeds. Bag ends were sealed with thermoplastic adhesive. Seed bags, each with seeds of one species, were placed in a 30 x 50 cm permeable sachet of sewn polyethylene mesh. Two *Pinus banksiana* cones and five rhizome cuttings from each of *Maianthemum canadense*, *Vaccinium myrtilloides* and *Arctostaphylos uva-ursi* were cut into 10 to 15 cm segments and placed in sachets. A total of 144 sachets were placed, 20 in each large stockpile and 16 in each small stockpile.

### Stockpile construction and instrumentation

Four replicates each of large and small stockpiles were built at four mine sites. At each site, three replicates were of coarse texture (sand) soil (orthic and eluviated eutric brunisol) and one was of fine texture (clay loam) soil (orthic gray luvisol). Stockpile construction began in October at sites 1, 2 and 3, and January the following year at site 4 due to operational logistics.

Large stockpiles were 36 x 20 m at base and 6 m high, big enough to accommodate operational size equipment. Small stockpiles were 4 x 15 m at base and 3 m high, simulating windrows found throughout the region. The upper 15 cm of topsoil was salvaged and windrowed with crawler tractors and large tracked excavators over 4 ha of logged forest at each mine site for each set of large and small stockpiles. The windrowed soil was hauled to placement areas and stockpiles were constructed in 1 m lifts.

In large stockpiles, sachets with seeds were placed at 6, 4, 2, 1 and 0.05 m depths and those containing cones and rhizome cuttings were placed at 6, 2 and 0.05 m depths. Soil probes were installed at 6, 4, 2, 1, 0.6 and 0.3 m depths. In small stockpiles, sachets were placed at 3, 2, 1 and 0.05 m depths and those containing cones and rhizome cuttings were placed at 3 and 0.05 m depths. Sachets were attached to 0.16 cm aircraft cable and orange snow fence strips for retrieval. Four sets of sachets were placed at each depth and were spread approximately 4 to 5 m apart in the centre of each stockpile to allow for multiple years of monitoring. The spacing was sufficient to prevent outside air affecting in situ sachets during extractions.

Soil probes were installed at 3, 1, 0.6 and 0.3 m depths. A total of 40 soil probes for soil water and 40 for soil temperature were installed; 5 of each were installed in each large stockpile and 4 in each small stockpile. A 1 m pit and trenched narrow path to the stockpile edge was dug with a tracked excavator. Campbell Scientific 107B soil and water temperature probes and CS616 soil volumetric water content reflectometers were installed in the pit face and wires strung in the trench, then backfilled. Sensors were connected to an automated data acquisition system and data were recorded every two hours for three years starting in January.

### Soil atmosphere

A total of 108 gas probes of nalgene teflon tubing (0.635 cm inside and 0.953 outside diameters) attached to an outside sealed polyvinyl chloride pipe for support with an air diffuser were installed in May of the first study year. Five probes were installed at 4, 3, 2, 1 and 0.3 m in large stockpiles and four at 3, 2, 1 and 0.3 m in small stockpiles, in 5 cm diameter holes augered by hand. One probe was inserted 15 cm above the bottom of each hole; 15 cm of sand was placed on top of it followed by 15 cm of bentonite chips and 1 L of water, then stockpiled soil. Soil gas was extracted from each probe from July through February at various times. The volume of gas in each tube was purged with a syringe prior to extracting 14 ml of gas into a 10 ml vacutainer, then stored at 4°C in the dark until analysis. Gas chromatographs for carbon dioxide, oxygen, nitrogen, methane and ethylene were determined.

### Seed viability and germination

Seeds were extracted in June of the first study year and February of the second using a tracked excavator by opening one end of the stockpile from the top downward. Seed bags were dried at room temperature. Viability was determined with 1% tetrazolium solution. Tetrazolium solutions, seed treatments, cutting methods and staining evaluations were according to the Association of Official Seed Analysts [21] and the International Seed Testing Association [22]. *Aralia nudicaulis* was not in the above protocols so seeds were placed between damp paper towel overnight, then cut longitudinally through the endosperm with a scalpel and stained with tetrazolium solution at 30°C in a dark oven for 18 h; viable seeds had bright red stained embryos and endosperm. Seeds that had germinated or rotted at the time of retrieval were classified as non-viable because they did not exhibit potential to germinate if stockpile material was spread on reclamation areas.

Germination and rhizome emergence were determined in a controlled growth chamber for 8 weeks, for pre stockpile and stored conditions on 25 seeds and 5 rhizome cuttings with 4 replicates each. Seeds were placed on damp seed germination blotter paper in sealable, plastic germination containers. Rhizome were planted 1 cm deep in containers filled with 5 cm of metromix. Seeds and rhizomes were watered twice weekly. Growth chambers were set to 28°C in light for 16 h and 15°C in dark for 9 h. Each week containers were randomly rotated and germination and rhizome emergence assessed. A seed was considered germinated when the first radical emerged and a rhizome was considered emerged when the first shoot emerged.

### Stockpile soil chemistry

Prior to salvaging, the upper 15 cm of topsoil was sampled with a shovel to provide baseline conditions for the stockpile material. Four samples of stockpiled soil were collected with a soil corer during each seed extraction, at each depth seed bags were extracted, from the face of the stockpile. Samples were analyzed according to Carter [23]. Saturation %, pH, electrical conductivity, sodium adsorption ratio, soluble cations (calcium, potassium, magnesium, sodium) and soluble anions (chloride, sulfate) were determined from saturated paste extract, total nitrogen by digestion with Devarda’s alloy and total carbon by combustion. Extractable cations (calcium, potassium, magnesium, sodium) and cation exchange capacity were determined with ammonium acetate at pH 7. Available phosphorus and potassium were determined by modified kelowna extraction, available ammonium and nitrate by extraction with 2 M potassium chloride, available copper, iron, zinc and manganese with diethylene triamine pentacetic acid, available and extractable boron by hot water extraction and available sulphate by monocalcium phosphate extraction. Particle size was determined by hydrometer.

### Statistical analyses

Separate one way fixed effects analysis of variance (ANOVA) for each stockpile size and extraction period (8 and 16 months) was used to determine whether stockpiling affected seed viability, seed germination, rhizome emergence and soil chemical properties by comparing response variables before and after stockpiling. Significant main effects were analyzed using least squares difference (LSD) post hoc test. Two way fixed effects ANOVA for each extraction period was used to determine effects of stockpile size and burial depth. Significant interaction effects were analyzed using one way ANOVAs to determine significant differences between depths for each stockpile size; if main effects were significant, differences were analyzed using LSD. Residuals from raw data were tested for normality with the Shapiro-Wilk test and heterogeneity of variances with Levene’s test. Data were rank transformed when variances of raw data were heterogeneous. Spearman’s rank correlation coefficient was used to compare strength of viability relationships of each species to soil gases and chemical properties known to affect seed germination, separately for large and small stockpiles. Analyses were conducted using SPSS 18.0; significance was at p = 0.05.

## Results and discussion

### Stockpiling effects on soil properties

**Fig 1:**
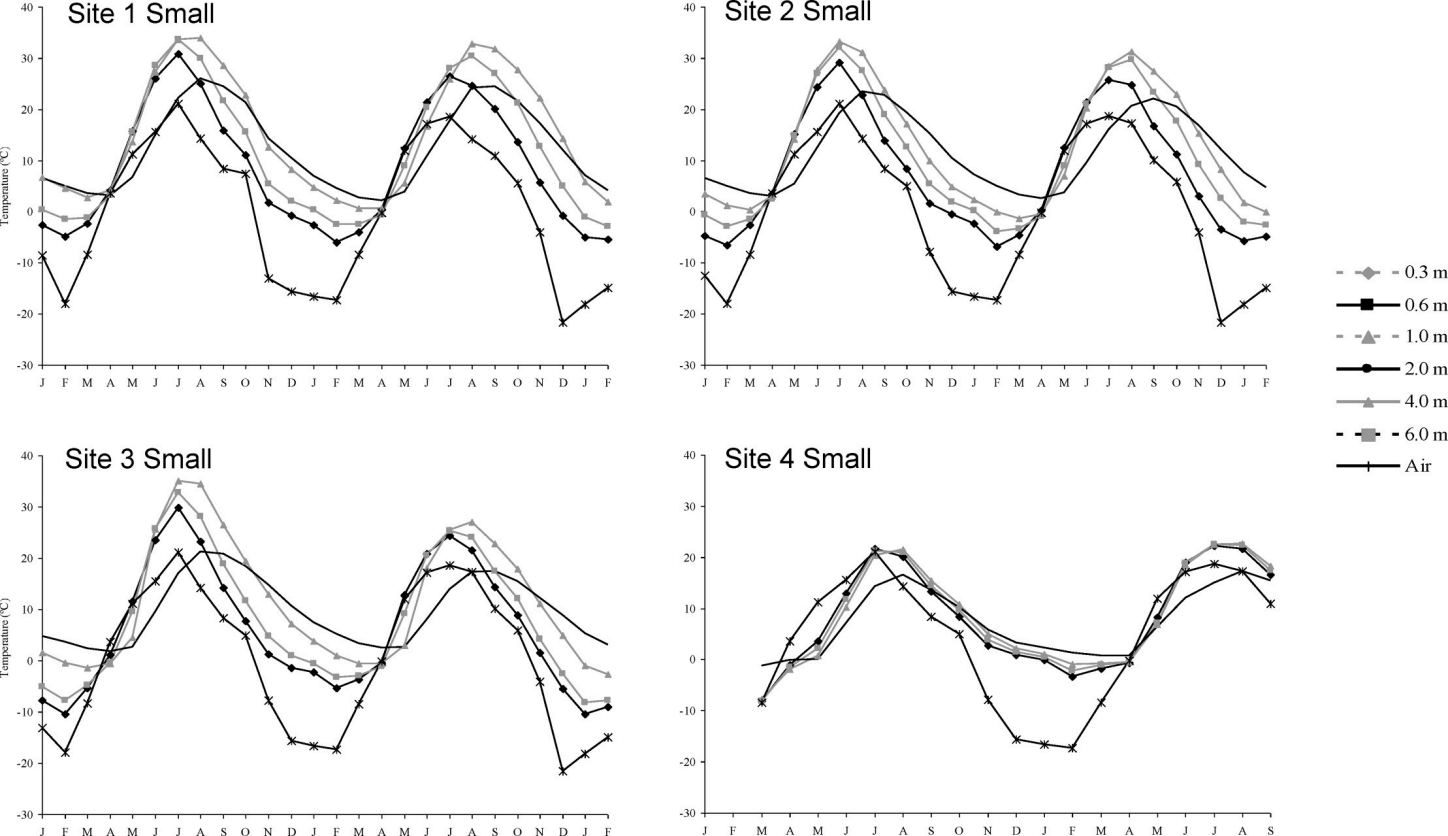
Monthly mean soil temperature at depth for small stockpiles between January of the first study year and February of the third.

**Fig 2:**
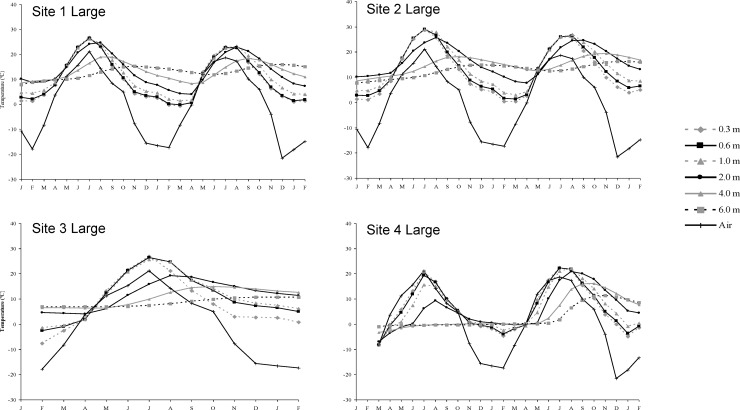
Monthly mean soil temperature at depth for large stockpiles between January of the first study year and February of the third.

**Fig 3:**
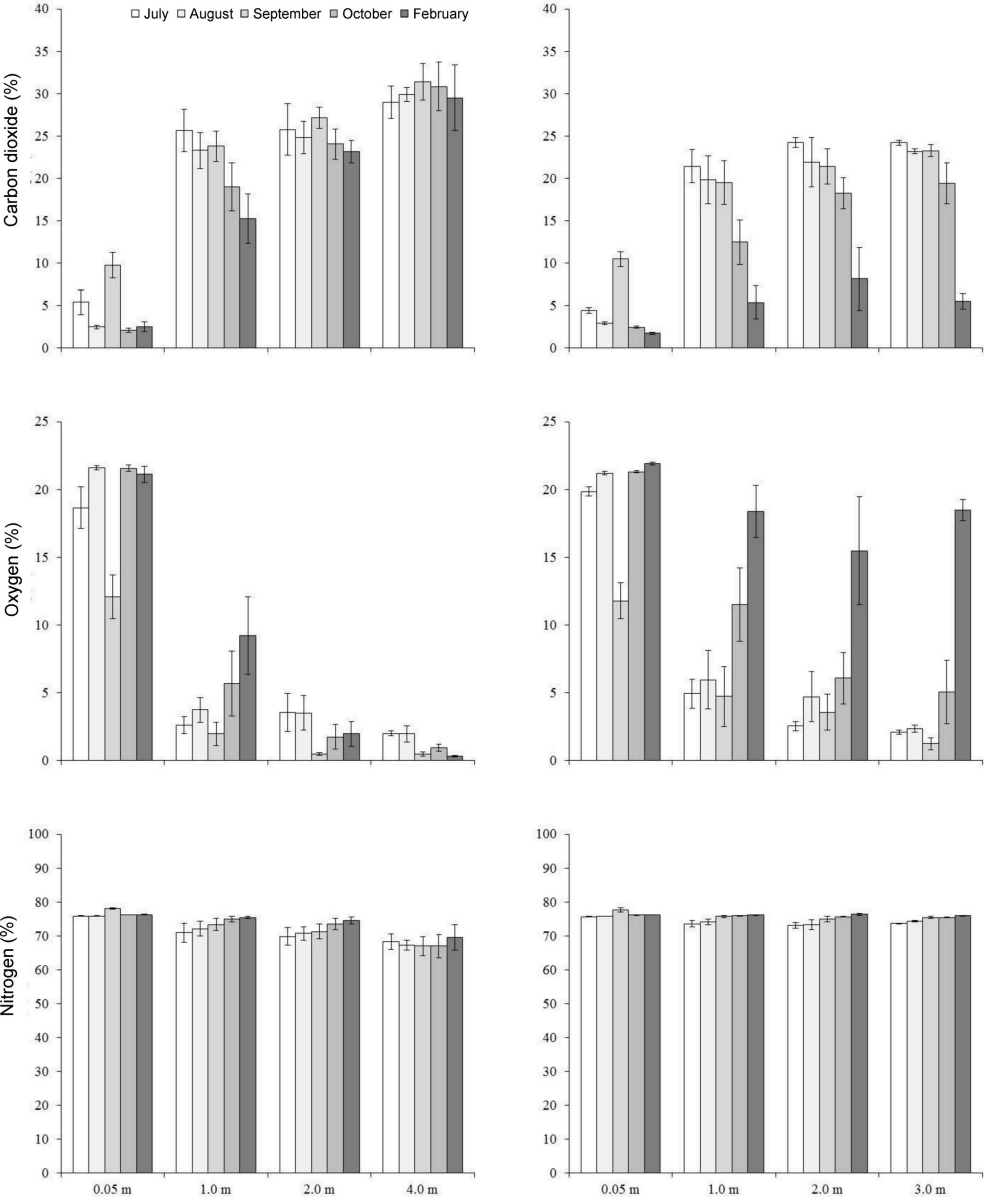
Monthly mean soil atmosphere carbon dioxide, oxygen and nitrogen concentrations by depth for large (left) and small stockpiles (right). n = 4.

**Fig 4:**
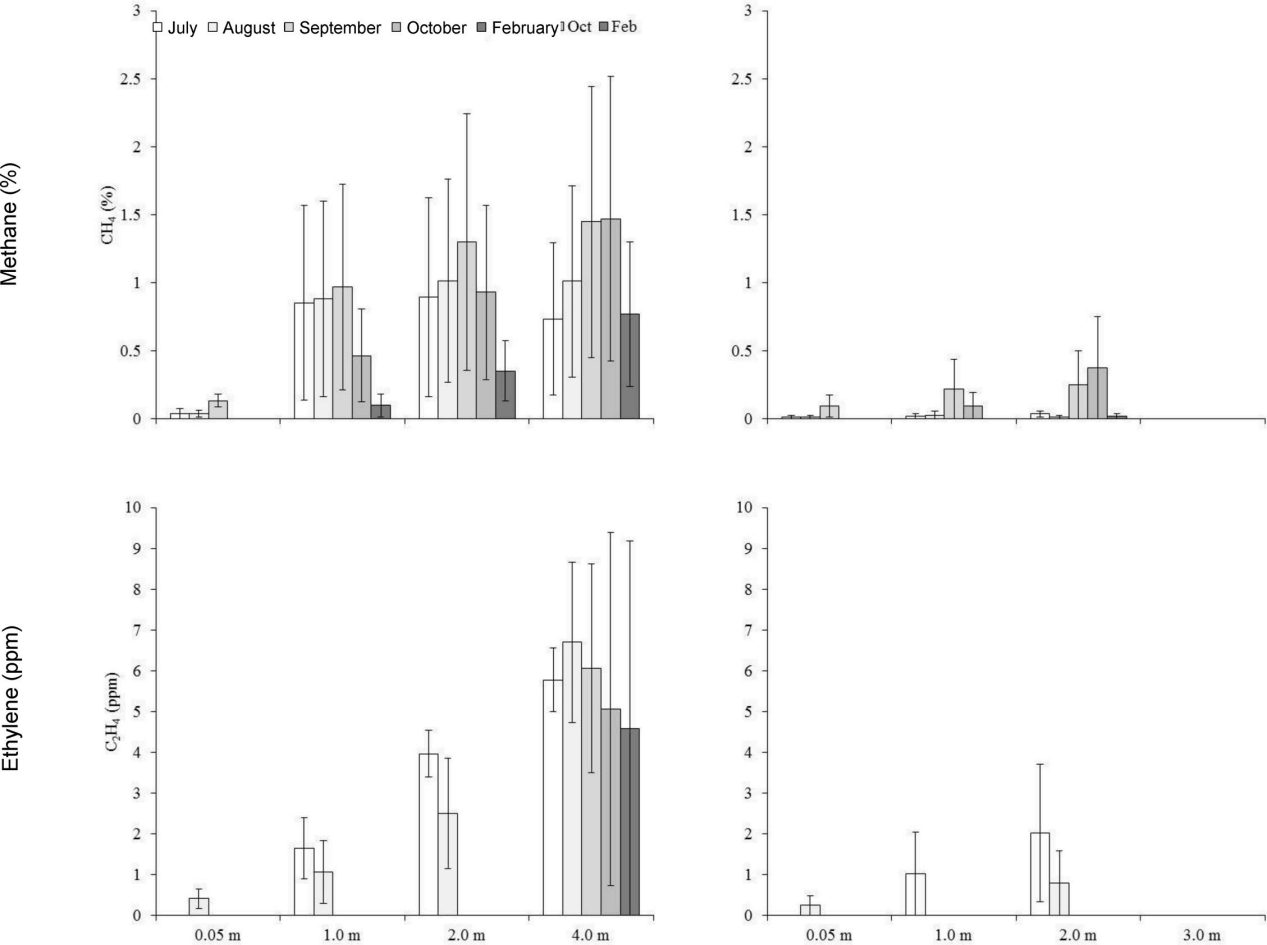
Monthly mean soil atmosphere methane and ethylene concentrations by depth for large (left) and small stockpiles (right). n = 4.

Stockpiling significantly changed some soil properties relative to undisturbed topsoil (Tables 1 and 2). Available ammonium increased significantly in large and small stockpiles. In large stockpiles there were significant increases in electrical conductivity, sodium adsorption ratio, soluble calcium and potassium and extractable manganese. Nitrate decreased with increased storage time in large stockpiles. Stockpile temperatures were generally higher than air temperature at all depths (Figs 1 and 2). Soil atmosphere carbon dioxide (Fig 3), ethylene and methane (Fig 4) increased with depth, whereas oxygen generally decreased with depth over time (Fig 3).

**Table 1:** Mean values for soil properties for burial depths after 16 months storage in large and small stockpiles.

Parameter	Control	Large Stockpile					Small Stockpile			
		0.05 m	1 m	2 m	4 m	6 m	0.05 m	1 m	2 m	3 m
Soil pH	5.6	5.9	5.9	6.0	5.5	5.4	5.7	5.7	5.7	5.6
	(0.2)	(0.4)	(0.5)	(0.4)	(0.4)	(0.3)	(0.2)	(0.2)	(0.2)	(0.3)
Electrical conductivity (dS/m)	0.4	0.7	0.5	0.7	1.5*	1.6*	0.6	0.4	0.4	0.3
	(0.0)	(0.1)	(0.0)	(0.3)	(0.5)	(0.5)	(0.1)	(0.0)	(0.0)	(0.0)
Sodium adsorption ratio	0.5	0.4^B^	0.5^B^	0.5^B^	0.3^B^	0.2*^B^	0.7 ^A^	0.6^A^	0.6^A^	0.7^A^
	(0.0)	(0.0)	(0.1)	(0.1)	(0.1)	(0.0)	(0.1)	(0.1)	(0.1)	(0.0)
Total carbon (%)	2.1	2.2	2.0	1.9	2.3	2.0	2.0	1.7	1.6	1.4
	(0.6)	(0.3)	(0.2)	(0.3)	(0.2)	(0.4)	(0.3)	(0.2)	(0.1)	(0.3)
Total nitrogen (%)	0.1	0.1	0.1	0.1	0.1	0.1	0.1	0.1	0.1	0.1
	(0.0)	(0.0)	(0.0)	(0.0)	(0.0)	(0.0)	(0.0)	(0.0)	(0.0)	(0.0)
Available nutrients (mg kg^-1^)										
Ammonium	3.1	5.7*^c^	12.0*^b^	15.0*^ab^	20.0*^a^	18.6*^ab^	6.7^c^	10.9*^bc^	13.5*^ab^	12.3*^abc^
	(0.3)	(0.6)	(3.4)	(3.7)	(3.2)	(4.7)	(2.2)	(3.0)	(4.1)	(2.3)
Nitrate	2.3	5.1^a^	6.20^a^	1.0^b^	0.6^b^	0.6^b^	1.3	3.7	1.7	0.9
	(0.2)	(1.5)	(2.2)	(0.2)	(0.1)	(0.0)	(0.5)	(2.2)	(0.8)	(0.3)
Phosphorous	17.9	18.5	19.8	20.3	25.5	31.5	22.0	21.3	26.3	18.0
	(3.1)	(3.9)	(3.7)	(4.6)	(4.7)	(5.0)	(3.9)	(4.0)	(4.4)	(7.2)
Potassium	95.8	101.0	89.3	96.8	101.8	107.0	137.8	82.8	101.0	67.8
	(19.9)	(29.4)	(13.2)	(20.8)	(30.9)	(31.1)	(54.8)	(15.9)	(20.1)	(12.8)
Sulphate	8.7	11.0	5.0	2.8*	5.0	6.3	9.0	6.3	4.3	3.5
	(1.2)	(4.1)	(0.4)	(0.9)	(0.4)	(0.9)	(2.9)	(1.7)	(0.3)	(0.5)
Soluble ions (mg L^-1^)										
Calcium	46.8	89.5^bc^	66.5^c^	93.0^c^	275.3*^ab^	295.3*^a^	60.8^cd^	40.0^c^	44.3^c^	31.3^d^
	(8.9)	(17.5)	(17.1)	(51.0)	(153.3)	(145.8)	(15.6)	(9.2)	(10.4)	(6.0)
Chloride	32.7	25.5	27.5	25.0	30.5	29.5	69.5	24.8	40.8	30.0
	(5.1)	(7.6)	(4.9)	(4.2)	(4.7)	(6.8)	(34.7)	(1.1)	(9.6)	(7.5)
Magnesium	12.0	24.8	16.5	27.8	74.8	76.3	16.0	11.5	12.3	8.8
	(2.8)	(4.9)	(4.8)	(17.6)	(45.6)	(41.3)	(3.3)	(3.2)	(4.4)	(2.3)

Data are mean with standard erroring brackets, n = 4. In rows * denotes LFH treatments significantly different from controls. In rows different letters denote significant differences; upper case letters between stockpile size and lower case letters between burial depths. Significant difference at p≤0.05.

**Table 2:** Mean values for soil properties for burial depths after 16 months storage in large and small stockpiles.

Parameter	Control	Large Stockpile					Small Stockpile			
		0.05 m	1 m	2 m	4 m	6 m	0.05 m	1 m	2 m	3 m
Potassium	19.7	32.3	25.3	29.8	51.3*	60.5*	51.0	16.8	18.3	12.3
	(5.0)	(14.2)	(6.4)	(3.6)	(6.0)	(12.6)	(38.3)	(4.3)	(1.7)	(3.1)
Sodium	13.4	16.5	16.8	15.5	16.5	14.3	20.0	15.5	17.5	17.3
	(1.5)	(3.0)	(2.0)	(3.3)	(4.6)	(2.1)	(3.1)	(0.6)	(2.1)	(2.9)
Sulphate	46.2	122.7	50.6	32.0	46.3	47.9	80.8	57.8	51.8	47.6
	(5.4)	(41.1)	(5.7)	(5.9)	(5.8)	(2.8)	(18.2)	(8.4)	(2.2)	(4.1)
Soluble ions (mg L^-1^); extractable boron (mg kg^-1^) and available micro nutrients (mg kg^-1^)										
Boron	0.4	0.4	0.3	0.3	0.3	0.3	0.5	0.4	0.4	0.2
	(0.2)	(0.2)	(0.2)	(0.1)	(0.2)	(0.1)	(0.2)	(0.2)	(0.2)	(0.1)
Copper	0.3	0.3	0.3	0.3	0.3	0.3	0.3	0.3	0.3	0.3
	(0.1)	(0.0)	(0.0)	(0.1)	(0.1)	(0.0)	(0.0)	(0.0)	(0.0)	(0.0)
Iron	51.7	47.3	79.0	146.0	99.3	82.0	62.3	58.3	74.5	83.5
	(6.2)	(13.1)	(17.0)	(61.8)	(11.7)	(14.1)	(12.2)	(12.6)	(3.8)	(7.9)
Manganese	53.3	17.5*^b^	46.6^b^	178.3*^a^	225.0*^a^	266.0*^a^	50.6	20.9	52.3	55.9
	(12.8)	(5.0)	(18.0)	(24.0)	(12.5)	(70.7)	(23.9)	(3.6)	(35.3)	(15.0)
Zinc	2.8	1.5*	1.4*	1.5*	2.1	1.8*	2.0*	1.7*	1.9*	1.3*
	(0.2)	(0.2)	(0.1)	(0.3)	(0.4)	(0.4)	(0.3)	(0.1)	(0.2)	(0.3)
Mean cation exchange capacity (meq 100 g-1) and exchangeable cations (meq 100 g-1)										
Cation exchange capacity	11.44	6.75	6.08	6.25	6.53	6.35	6.88	6.38	6.65	5.18
	(3.84)	(1.98)	(1.62)	(1.80)	(1.69)	(2.32)	(1.70)	(1.25)	(2.26)	(1.31)
Calcium	5.3	5.15	4.08	4.6	4.58	3.98	4.53	3.88	4.03	2.78
	(2.16)	(1.79)	(1.48)	(2.10)	(1.90)	(1.76)	(1.47)	(1.23)	(1.43)	(0.66)
Magnesium	1.05	0.98	0.68	0.75	0.73	0.65	0.95	0.75	0.8	0.58
	(0.61)	(0.71)	(0.50)	(0.60)	(0.57)	(0.50)	(0.55)	(0.50)	(0.52)	(0.43)
Potassium	0.2	0.15	0.15	0.2	0.25	0.18	0.33	0.13	0.18	0.05
	(0.10)	(0.10)	(0.05)	(0.08)	(0.05)	(0.10)	(0.15)	(0.08)	(0.10)	(0.05)
Sodium	0.01	0.07	0.07	0.13	0.13	0.13	0	0	0.05	0
	(0.01)	(0.06)	(0.06)	(0.12)	(0.12)	(0.12)	(0.00)	(0.00)	(0.05)	(0.00)

Data are mean with standard erroring brackets, n = 4. In rows * denotes LFH treatments significantly different from controls. In rows different letters denote significant differences between burial depths. Significant difference at p≤0.05.

Soil impacts from stockpiling support results from other research [8, 24]. Extreme anaerobic conditions developed in large stockpiles shortly after construction and persisted, but occurred over time at depth in small stockpiles. The fine texture, large stockpile was most anaerobic. Anaerobic conditions promote reduction of soil elements used as alternative electron acceptors, including nitrate, manganese dioxide, hydrated iron oxide, sulphate, carbon dioxide and water, which increases ammonium, manganese, iron, hydrogen sulphide and methane; nitrate, manganese dioxide and hydrated iron oxdide reduction can occur in hypoxic conditions where carbon dioxide and sulphate reduction occur under extreme anaerobic conditions [25].

Rate and magnitude of change from aerobic to anaerobic states are affected by porosity, organic matter, water content and temperature [26], which regulate microbial activity. Except for organic matter, stockpile size and construction time, soil depth and length of storage greatly influence these factors. Changes to air filled porosity likely caused the most abrupt change in the large stockpiles with increasing depths below 1 m. Increased equipment traffic and large amounts of piled soil compact soil, reducing aeration and air filled porosity. With limited oxygen, aerobic respiration quickly switches to anaerobic, fueled by organic matter in stockpiled soil. How fast the change occurs depends on temperature. Stockpiles that initially became most anaerobic were constructed in fall or when soil temperature was above 0°C below 1 m; the stockpile with the most total carbon was most anaerobic.

Small stockpiles became anaerobic over time, but not as much as large stockpiles. They were much less compact and the higher porosity would have allowed more oxygen in the stockpile to support aerobic respiration for a longer time. Increased aerobic respiration in spring and early summer would have decreased oxygen, leading to anaerobic respiration. In June, the surface, 0.6 and 1.0 m depths were 10°C warmer than air temperature and by August all depths were 10 to 20°C warmer than air temperature. Piling topsoil with an abundant source of organic carbon is similar to creating a compost pile. Factors such as temperature and water content influence anaerobicity and while the winter constructed stockpile was initially less anaerobic than the fall constructed one, when snow melted, increased water caused the winter constructed stockpile to become much more anaerobic (water data not shown).

Substantial changes in soil chemistry in large stockpiles could have consequences for land reclamation. Ammonium can be lost by leaching, volatilization or run off once material is spread. Harris et al. [27] show stockpiling topsoil increased lability of organic nitrogen making it more susceptible to mineralization, increasing potential for loss. Increased major cations deep in the stockpiles likely result from leaching and desorption from exchange sties. Ion leaching is negligible as highly mobile anions such as chloride did not increase much with depth. High concentrations of ammonium in soil likely displaced potassium on exchange sites, increasing soluble potassium [28]. High concentrations of carbon dioxide increase hydrogen ions when carbon dioxide is dissolved in water and hydrogen ions displace calcium from exchange sites making it more soluble. Increased soluble calcium and magnesium could have formed strong complexes with zinc, reducing its concentration. Increased availability of these elements along with increased concentrations of more mobile forms of manganese and iron make them more susceptible to leaching from the stockpile and after spreading on a reclamation landscape. Long term impacts to nutrient availability using spread stockpiled soil is unknown; a large pool of nutrients will likely be available shortly after soil placement and if nutrients are not taken up by plants, most could be lost to leaching, run off and volatilization.

### Stockpiling effects on seed viability and germination

Stockpiling rapidly reduced seed viability and germination and rhizome emergence for most species, with continued reductions over time (Tables 3–12). Stockpiling resulted in a significant decline (up to 100%) in seed viability of 24 of the 27 species in small and large stockpiles at most burial depths after 8 and 16 months. There were few significant differences with stockpile size and burial depth; however, after 8 months there were greater declines in viability below 1 m in large stockpiles. After 8 months rhizome emergence declined more than 80% for *Arctostaphylos uva-ursi*, *Maianthemum canadense* and *Vaccinium myrtilloides*; after 16 months no rhizomes survived. Fall construction resulted in greater seed viability declines than winter construction; declines were greater in the fine texture stockpile than the coarse texture stockpiles for most species.

**Table 3:** Mean percent seed viability of grasses for burial depths after 8 months of storage in large and small stockpiles.

Species	Control	Large Stockpile					Small Stockpile			
		0.05 m	1 m	2 m	4 m	6 m	0.05 m	1 m	2 m	3 m
*Agropyron trachycaulum*	73	0*	48	38*	15*	26*	31	40	39	43
	(5.5)	(0.0)	(9.4)	(13.1)	(15.0)	(12.5)	(17.9)	(8.5)	(13.7)	(4.1)
*Bromus ciliatus*	58	15*	41	15*	11*	10*	38	38	50	43
	(5.8)	(13.7)	(6.2)	(10.0)	(11.0)	(10.0)	(9.0)	(8.4)	(11.4)	(4.4)
*Carex anea*	94	87	95	75	24	75	88	93	86	90
	(2.6)	(6.4)	(2.5)	(17.3)	(24.0)	(18.4)	(4.9)	(2.5)	(4.8)	(2.6)
*Elymus innovatus*	54	3*	7*	3*	11*	9*	1*	0*	8*	18*
	(8.7)	(3.0)	(7.0)	(3.0)	(11.0)	(7.7)	(1.0)	(0.0)	(8.0)	(5.3)
*Oryzopsis pungens*	91	58	59	23	21	24	68	39*	83	84
	(4.1)	(11.8)	(19.8)	(19.2)	(21.0)	(21.4)	(6.7)	(14.8)	(1.9)	(2.3)

Data are mean and (standard error), n = 4. In rows * denotes LFH treatments significantly different from the control. Significant difference at p≤0.05.

**Table 4:** Mean percent seed viability of grasses for burial depths after 16 months of storage in large and small stockpiles.

Species	Control	Large Stockpile					Small Stockpile			
		0.05 m	1 m	2 m	4 m	6 m	0.05 m	1 m	2 m	3 m
*Agropyron trachycaulum*	73	0*	11*	14*	0*	0*	0*	4*	4*	1*
	(5.5)	(0.0)	(7.5)	(14.0)	(0.0)	(0.0)	(0.0)	(4.0)	(4.0)	(1.0)
*Bromus ciliatus*	58	0*	10*	7*	0*	0*	0*	10*	0*	0*
	(5.8)	(0.0)	(8.7)	(7.0)	(0.0)	(0.0)	(0.0)	(10.0)	(0.0)	(0.0)
*Carex anea*	94	76*	90	26*	0*	16*	88	98	57	48
	(2.6)	(4.9)	(3.8)	(17.5)	(0.0)	(14.0)	(5.9)	(2.0)	(23.0)	(24.7)
*Elymus innovatus*	54	0*	0*	6*	0*	0*	0*	0*	0*	0*
	(8.7)	(0.0)	(0.0)	(6.0)	(0.0)	(0.0)	(0.0)	(0.0)	(0.0)	(0.0)
*Oryzopsis pungens*	91	23*	30*	0*	0*	0*	34*	9*	11*	0*
	(4.1)	(4.1)	(10.5)	(0.0)	(0.0)	(0.0)	(11.0)	(5.7)	(6.4)	(0.0)

Data are mean and (standard error), n = 4. In rows * denotes LFH treatments significantly different from the control. Significant difference at p≤0.05.

**Table 5:** Mean percent seed viability of herbaceous plants for burial depths after 8 months of storage in large and small stockpiles.

Species	Control	Large Stockpile					Small Stockpile			
		0.05 m	1 m	2 m	4 m	6 m	0.05 m	1 m	2 m	3 m
*Anemone multifida*	63	16	54	20	19	0	39	23	45	51
	(4.4)	(5.9)	(14.8)	(17.4)	(19.0)	(12.5)	(12.9)	(9.0)	(16.8)	(3.0)
*Anemone patens*	70	18*	23*	13*	7*	6*	26*	9*	16*	14*
	(2.0)	(6.2)	(10.9)	(13.0)	(7.0)	(6.0)	(9.6)	(5.3)	(11.0)	(4.8)
*Aralia nudicaulis*	59	10*	6*	4*	4*	2*	15*	14*	10*	11*
	(5.7)	(1.2)	(3.5)	(4.0)	(4.0)	(2.0)	(1.9)	(6.0)	(4.2)	(1.9)
*Cornus canadensis*	82	82	86	56	25	55*	81	80	82	87
	(2.6)	(1.2)	(1.2)	(18.8)	(21.2)	(18.4)	(4.4)	(0.0)	(1.2)	(1.9)
*Dracocephalum parviflorum*	97	98	97	92	86	94	94	99	98	98
	(1.0)	(2.0)	(1.0)	(3.7)	(4.8)	(2.0)	(2.6)	(1.0)	(1.2)	(2.0)
*Fragaria virginiana*	88	66	69	30	20	40	75.5	72	66	78
	(2.3)	(12.9)	(15.3)	(19.9)	(20.0)	(22.0)	(9.5)	(8.2)	(18.2)	(3.8)
*Geranium bicknellii*	97	78*	77*	79*	84*	84*	79*	56*	78*	84*
	(1.9)	(2.6)	(11.0)	(5.7)	(3.7)	(1.6)	(1.0)	(5.7)	(7.4)	(1.6)
*Rubus pubescens*	87	62*^a^	46*^b^	12*^c^	6*^d^	16*^d^	68^a^	44^b^	32*^c^	56*^a^
	(3.4)	(5.8)	(9.9)	(9.5)	(6.0)	(9.9)	(2.8)	(13.5)	(12.5)	(5.9)
*Vicia americana*	84	30*	60	43*	20*	41*	20	68	54	59
	(4.3)	(7.4)	(12.1)	(7.5)	(9.1)	(13.7)	(7.8)	(18.8)	(21.5)	(10.0)
*Maianthemum canadense*	90	30*	84	26*	21*	23*	82	64	68	68
	(2.6)	(10.9)	(7.1)	(21.0)	(21.0)	(21.7)	(3.5)	(21.5)	(22.7)	(22.7)
*Potentilla tridentata*	59	31*	30*	11*	10*	12*	31	33	28	35
	(4.4)	(3.4)	(6.6)	(6.2)	(10.0)	(12.0)	(1.9)	(9.0)	(9.9)	(7.5)

Data are mean and (standard error), n = 4. In rows * denotes LFH treatments significantly different from the control. In rows different letters denote significant differences between burial depths. Significant difference at p≤0.05.

**Table 6:** Mean percent seed viability of herbaceous plants for burial depths after 16 months of storage in large and small stockpiles.

Species	Control	Large Stockpile					Small Stockpile			
		0.05 m	1 m	2 m	4 m	6 m	0.05 m	1 m	2 m	3 m
*Anemone multifida*	63	10*	40	13*	0*	0*	25*	2*	21*	0*
	(4.4)	(6.6)	(13.6)	(13.0)	(0.0)	(0.0)	(13.0)	(2.0)	(13.4)	(0.0)
*Anemone patens*	70	0*	6*	4*	0*	0*	1*	2*	1*	0*
	(2.0)	(0.0)	(4.8)	(4.0)	(0.0)	(0.0)	(1.0)	(2.0)	(1.0)	(0.0)
*Aralia nudicaulis*	59	8*	5*	8*	0*	0*	4*	6*	0*	0*
	(5.7)	(4.9)	(5.0)	(8.0)	(0.0)	(0.0)	(4.0)	(3.8)	(0.0)	(0.0)
*Cornus canadensis*	82	66^a^	34*^b^	6*^c^	6*^d^	13*^d^	67*^a^	37*^b^	22*^c^	0*^d^
	(2.6)	(3.5)	(13.1)	(3.8)	(6.0)	(13.0)	(5.3)	(14.7)	(8.1)	(0.0)
*Dracocephalum parviflorum*	97	97^a^	99^a^	97^a^	89*^b^	96^ab^	96^a^	98^a^	96^a^	98^a^
	(1.0)	(1.0)	(1.0)	(1.9)	(3.4)	(0.0)	(1.6)	(1.2)	(1.6)	(1.2)
*Fragaria virginiana*	88	25*^a^	61^a^	14*	5*^b^	3*^b^	44.5*^a^	43*^a^	3*^b^	1*^b^
	(2.3)	(5.0)	(11.5)	(14.0)	(5.0)	(3.0)	(10.7)	(17.4)	(3.0)	(1.0)
*Geranium bicknellii*	97	86^a^	33^b^	79^a^	83^a^	88^a^	76^a^	39*^b^	55^a^	68^a^
	(1.9)	(1.2)	(22.4)	(6.8)	(3.0)	(5.3)	(11.4)	(18.4)	(22.6)	(22.7)
*Rubus pubescens*	87	50	13*	2*	0*	1*	73	13*	9*	2*
	(3.4)	(23.1)	(13.0)	(2.0)	(0.0)	(1.0)	(4.4)	(13.0)	(9.0)	(2.0)
*Vicia americana*	84	15*	30*	12*	14*	35*	18*	39*	18*	43*
	(4.3)	(8.7)	(11.6)	(9.5)	(3.5)	(13.9)	(14.3)	(15.3)	(8.4)	(15.6)
*Maianthemum canadense*	90	46	31*	15*	0*	0*	81	26*	0*	0*
	(2.6)	(24.4)	(17.3)	(15.0)	(0.0)	(0.0)	(6.2)	(22.2)	(0.0)	(0.0)
*Potentilla tridentata*	59	20*	7*	10*	0*	0*	14*	6*	3*	0*
	(4.4)	(3.7)	(3.4)	(10.0)	(0.0)	(0.0)	(3.5)	(3.8)	(1.9)	(0.0)

Data are mean and (standard error), n = 4. In rows * denotes LFH treatments significantly different from the control. In rows different letters denote significant differences between burial depths. Significant difference at p≤0.05.

**Table 7:** Mean percent seed viability of woody plants for burial depths after 8 months of storage in large and small stockpiles.

Species	Control	Large Stockpile					Small Stockpile			
		0.05 m	1 m	2 m	4 m	6 m	0.05 m	1 m	2 m	3 m
*Alnus crispa*	18	1*	4*	0*	2*	2*	6	4	9	3
	(2.0)	(1.0)	(2.3)	(0.0)	(2.0)	(2.0)	(2.6)	(4.0)	(5.7)	(1.9)
*Arctostaphylos uva-ursi*	60	42	59	49	23	49	46	65	44*	50
	(4.3)	(8.1)	(11.1)	(13.5)	(17.0)	(13.9)	(7.7)	(3.4)	(4.3)	(2.0)
*Prunus pensylvanica*	88	86	49	28*	22*	28*	86	68	62	78
	(2.8)	(2.6)	(10.8)	(20.3)	(22.0)	(20.8)	(3.5)	(17.5)	(21.1)	(3.5)
*Ribes hudsonianum*	98	86*	94	58	17*	61*	93	88	72	93
	(1.2)	(2.6)	(2.0)	(24.3)	(17.0)	(20.4)	(1.9)	(4.3)	(24.1)	(3.0)
*Rosa acicularis*	56	31	24	34	18	34	36	33	31	37
	(8.2)	(4.7)	(5.9)	(11.6)	(18.0)	(12.4)	(4.9)	(4.4)	(9.0)	(3.0)
*Rubus idaeus*	92	86	85	47*	22*	57*	78*	67*	64*	80*
	(2.8)	(3.8)	(1.9)	(18.6)	(20.7)	(19.6)	(2.6)	(11.7)	(20.0)	(2.3)
*Shepherdia canadensis*	93	74^a^	25*^a^	20*^b^	8*^b^	12*^b^	87^a^	74*^a^	25*^b^	49*^a^
	(1.9)	(9.6)	(23.7)	(20.0)	(8.0)	(12.0)	(2.5)	(9.0)	(19.8)	(13.7)
*Vaccinium myrtilloides*	77	45	37*	31*	14*	24*	64*	31*	11*	15*
	(5.0)	(4.4)	(10.0)	(15.2)	(14.0)	(16.1)	(4.9)	(2.5)	(3.8)	(1.9)
*Vaccinium vitis- idea*	89	83	70	34	22	31	83	82	66	86
	(4.1)	(3.4)	(12.7)	(20.7)	(22.0)	(19.5)	(1.9)	(2.0)	(22.1)	(2.6)
*Virburnum edule*	88	86	79	66	40	73	87	63	65	86
	(1.6)	(1.2)	(7.2)	(14.1)	(14.9)	(7.2)	(5.3)	(21.1)	(9.8)	(6.2)
*Pinus banksiana*	87	57	-	57	-	25	75	-	-	68
	(2.5)	(15.4)	-	(15.4)	-	(25.0)	(3.4)	-	-	(11.2)

Data are mean and (standard error), n = 4. In rows * denotes LFH treatments significantly different from the control. In rows different letters denote significant differences between burial depths. Significant difference at p≤0.05.

**Table 8:** Mean percent seed viability of woody plants for burial depths after 16 months of storage in large and small stockpiles.

Species	Control	Large Stockpile					Small Stockpile			
		0.05 m	1 m	2 m	4 m	6 m	0.05 m	1 m	2 m	3 m
*Alnus crispa*	18	4*	1*	0*	0*	0*	4*	0*	0*	0*
	(2.0)	(2.8)	(1.0)	(0.0)	(0.0)	(0.0)	(2.8)	(0.0)	(0.0)	(0.0)
*Arctostaphylos uva-ursi*	60	13*	0*	0*	0*	0*	17*	6*	0*	0*
	(4.3)	(13.0)	(0.0)	(0.0)	(0.0)	(0.0)	(17.0)	(6.0)	(0.0)	(0.0)
*Prunus pensylvanica*	88	82^a^	25*^b^	12*^bc^	2*^c^	0*^c^	49*^a^	22*^b^	11*^bc^	3*^c^
	(2.8)	(2.6)	(13.7)	(10.7)	(2.0)	(0.0)	(16.8)	(12.9)	(4.1)	(3.0)
*Ribes hudsonianum*	98	21*	0*	0*	0*	0*	4*	0*	0*	0*
	(1.2)	(9.1)	(0.0)	(0.0)	(0.0)	(0.0)	(2.8)	(0.0)	(0.0)	(0.0)
*Rosa acicularis*	56	0*	14*	0*	11*	6*	14*	6*	5*	6*
	(8.2)	(0.0)	(8.7)	(0.0)	(11.0)	(6.0)	(8.2)	(6.0)	(5.0)	(3.5)
*Rubus idaeus*	92	67*^a^	64^a^	21*^b^	11*^b^	0*^b^	78^a^	60^a^	20*^b^	24*^ab^
	(2.8)	(9.4)	(21.4)	(21.0)	(11.0)	(0.0)	(2.6)	(20.1)	(20.0)	(18.0)
*Shepherdia canadensis*	93	49	10*	9*	0*	0*	47*	0*	0*	0*
	(1.9)	(14.8)	(6.0)	(5.3)	(0.0)	(0.0)	(14.8)	(0.0)	(0.0)	(0.0)
*Vaccinium myrtilloides*	77	39*	18*	15*	0*	0*	34*	12*	0*	6*
	(5.0)	(7.0)	(10.5)	(15.0)	(0.0)	(0.0)	(2.6)	(12.0)	(0.0)	(3.5)
*Vaccinium vitis- idea*	89	64*	43*	16*	0*	0*	49*	42*	5*	0*
	(4.1)	(3.3)	(19.0)	(16.0)	(0.0)	(0.0)	(8.7)	(8.7)	(5.0)	(0.0)
*Virburnum edule*	88	58*^a^	20*^b^	5*^b^	1*^b^	18*^b^	72*^a^	20*^b^	14*^b^	6*^b^
	(1.6)	(6.6)	(17.4)	(5.0)	(1.0)	(18.0)	(4.0)	(20.0)	(14.0)	(6.0)
*Pinus banksiana*	87	49.3*	-	2*	-	0*	62*	-	-	0*
	(2.5)	(19.6)	-	(2.0)	-	(0.0)	(13.3)	-	-	(0.0)

Data are mean and (standard error), n = 4. In rows * denotes LFH treatments significantly different from the control. In rows different letters denote significant differences between burial depths. Significant difference at p≤0.05.

**Table 9:** Mean percent germination of grasses for burial depths after 8 months of storage in large and small stockpiles.

Species	Control	Large Stockpile					Small Stockpile			
		0.05 m	1 m	2 m	4 m	6 m	0.05 m	1 m	2 m	3 m
*Agropyron trachycaulum*	72	0*	42	13*	15*	18*	18*	33*	35*	40*
	(6.7)	(0.0)	12.49	(13.0)	(15.0)	(14.3)	(11.8)	(3.0)	(11.7)	(4.0)
*Bromus ciliatus*	58	1*^B^	35^B^	15*^B^	10*^B^	10*^B^	38^A^	36^A^	47^A^	37^A^
	(2.6)	(1.0)	(11.9)	(10.0)	(10.0)	(10.0)	(9.0)	(10.2)	(15.7)	(8.2)
*Carex anea*	93	83	95	73	22	72	91	91	70	71
	(3.0)	(6.6)	(2.5)	(16.8)	(22.0)	(21.4)	(4.1)	(3.4)	(17.0)	(17.3)
*Elymus innovatus*	55	3*	6*	3*	11*	9*	0*	0*	8*	17*
	(5.3)	(3.0)	(3.8)	(3.0)	(11.0)	(7.7)	(0.0)	(0.0)	(8.0)	(5.5)
*Oryzopsis pungens*	66	40*^a^	5*^b^	10*^b^	5*^b^	7*^b^	31*^a^	20*^b^	27*^b^	41*^a^
	(2.6)	(2.8)	(3.0)	(6.6)	(5.0)	(7.0)	(5.3)	(6.3)	(9.1)	(7.0)

Data are mean and (standard error), n = 4. In rows * denotes LFH treatments significantly different from the control. In rows different letters denote significant differences, upper case letters between stockpile size and lower case letters between burial depths. Significant difference at p≤0.05.

**Table 10:** Mean percent germination of herbaceous plants for burial depths after 8 months of storage in large and small stockpiles.

Species	Control	Large Stockpile					Small Stockpile			
		0.05 m	1 m	2 m	4 m	6 m	0.05 m	1 m	2 m	3 m
*Anemone multifida*	38	7	30	10	8	10	39	4	26	23
	(11.8)	(3.0)	(12.7)	(10.0)	(8.0)	(10.0)	(14.4)	(2.8)	(10.6)	(7.2)
*Anemone patens*	51	9*	19*	1*	1*	2*	17*	12*	12*	13*
	(5.5)	(7.7)	(7.5)	(1.0)	(1.0)	(2.0)	(7.4)	(5.2)	(12.0)	(4.4)
*Aralia nudicaulis*	0	0	0	0	0	0	0	0	0	0
	(0.0)	(0.0)	(0.0)	(0.0)	(0.0)	(0.0)	(0.0)	(0.0)	(0.0)	(0.0)
*Cornus canadensis*	0	0	1	0	0	0	0	0	0	0
	(0.0)	(0.0)	(1.0)	(0.0)	(0.0)	(0.0)	(0.0)	(0.0)	(0.0)	(0.0)
*Dracocephalum parviflorum*	9	3	7	2*	0*	0*	2	1	1	4
	(3.4)	(1.0)	(1.9)	(2.0)	(0.0)	(0.0)	(1.2)	(1.0)	(1.0)	(2.3)
*Fragaria virginiana*	62	48	55	18*	17*	16*	71	54	51	51
	(8.7)	(9.4)	(13.9)	(13.2)	(15.7)	(9.2)	(5.3)	(9.3)	(16.2)	(5.7)
*Geranium bicknellii*	5	2	0	0	0	0	0	0	0	1
	(3.8)	(2.0)	(0.0)	(0.0)	(0.0)	(0.0)	(0.0)	(0.0)	(0.0)	(1.0)
*Rubus pubescens*	0	0	0	0	0	0	2	0	0	0
	(0.0)	(0.0)	(0.0)	(0.0)	(0.0)	(0.0)	(2.0)	(0.0)	(0.0)	(0.0)
*Vicia americana*	24	0*	3*	0*	1*	2*	0*	1*	0*	4*
	(4.3)	(0.0)	(3.0)	(0.0)	(1.0)	(2.0)	(0.0)	(1.0)	(0.0)	(1.6)
*Maianthemum canadense*	0	0	0	0	0	0	0	0	0	0
	(0.0)	(0.0)	(0.0)	(0.0)	(0.0)	(0.0)	(0.0)	(0.0)	(0.0)	(0.0)
*Potentilla tridentata*	21	14*^a^	5*^b^	2*^b^	1*^b^	3*^b^	18^a^	4*^b^	7*^b^	9*^a^
	(5.5)	(2.6)	(1.0)	(1.2)	(1.0)	(3.0)	(3.8)	(1.6)	(4.4)	(2.5)

Data are mean and (standard error), n = 4. In rows * denotes LFH treatments significantly different from the control. In rows different letters denote significant differences between burial depths. Significant difference at p≤0.05.

**Table 11:** Mean percent germination of woody plants for burial depths after 8 months of storage in large and small stockpiles.

Species	Control	Large Stockpile					Small Stockpile			
		0.05 m	1 m	2 m	4 m	6 m	0.05 m	1 m	2 m	3 m
*Alnus crispa*	13	5*	4*	3*	2*	1*	6	2	6	3
	(1.9)	(1.9)	(2.3)	(3.0)	(2.0)	(1.0)	(3.5)	(2.0)	(6.0)	(1.9)
*Arctostaphylos uva-ursi*	0	0	0	0	0	0	0	0	0	0
	(0.0)	(0.0)	(0.0)	(0.0)	(0.0)	(0.0)	(0.0)	(0.0)	(0.0)	(0.0)
*Prunus pensylvanica*	0	0	0	0	0	0	0	0	0	0
	(0.0)	(0.0)	(0.0)	(0.0)	(0.0)	(0.0)	(0.0)	(0.0)	(0.0)	(0.0)
*Ribes hudsonianum*	16	13	8	5	0*	1*	5	6	0*	1*
	(9.7)	(4.4)	(4.3)	(3.8)	(0.0)	(1.0)	(1.9)	(1.2)	(0.0)	(1.0)
*Rosa acicularis*	0	0	0	0	0	0	0	0	0	0
	(0.0)	(0.0)	(0.0)	(0.0)	(0.0)	(0.0)	(0.0)	(0.0)	(0.0)	(0.0)
*Rubus idaeus*	0	3	1	0	0	0	0	0	0	1
	(0.0)	(3.0)	(1.0)	(0.0)	(0.0)	(0.0)	(0.0)	(0.0)	(0.0)	(1.0)
*Shepherdia canadensis*	0	5*	1	0	0	0	3	3	0	0
	(0.0)	(2.5)	(1.0)	(0.0)	(0.0)	(0.0)	(3.0)	(3.0)	(0.0)	(0.0)
*Vaccinium myrtilloides*	41	20*	20*	12*	3*	11*	13*	11*	5*	8*
	(2.5)	(5.7)	(8.2)	(6.3)	(3.0)	(6.4)	(3.0)	(3.0)	(1.9)	(1.6)
*Vaccinium vitis-idea*	32	2*	10*	4*	0*	3*	2	3*	3*	7*
	(3.7)	(1.2)	(4.2)	(4.0)	(0.0)	(1.9)	(2.0)	(1.9)	(3.0)	(1.9)
*Virburnum edule*	0	0	0	0	0	0	0	0	0	0
	(0.0)	(0.0)	(0.0)	(0.0)	(0.0)	(0.0)	(0.0)	(0.0)	(0.0)	(0.0)
*Pinus banksiana*	82	53	-	47*	-	20*	67*	-	-	55*
	(3.8)	(15.8)	-	(14.5)	-	(20.0)	(3.4)	-	-	(9.3)

Data are mean and (standard error), n = 4. In rows * denotes LFH treatments significantly different from the control. Significant difference at p≤0.05.

**Table 12:** Mean percent rhizome emergence of various plants for burial depths after 8 months of storage in large and small stockpiles.

Species	Control	Large Stockpile			Small Stockpile	
		0.05 m	2 m	6 m	0.05 m	3 m
*Arctostaphylos uva-ursi*	85	5*	0*	0*	0*	0*
	(9.5)	(5.0)	(0.0)	(0.0)	(0.0)	(0.0)
*Maianthemum canadense*	100	25*	10*	5*	15*	10*
	(0.0)	(9.6)	(10.0)	(5.0)	(5.0)	(5.8)
*Vaccinium myrtilloides*	95	35*	5*	15*	30*	35*
	(5.0)	(17.1)	(5.0)	(9.6)	(17.3)	(12.6)

Data are mean and (standard error), n = 4. In rows * denotes LFH treatments significantly different from the control. Significant difference at p≤0.05.

Seed viability and germination did not differ significantly between extraction times and burial depths for most species, although trends of decreasing viability over time with increasing depth were clear. Other studies found stockpiling reduced native seed viability over a short time [14, 12, 29]. Rivera et al. [29] found storage of topsoil in Spain significantly decreased seed viability and germination of 10 grass species within 6 months and with increased burial depth. Rockich et al. [12] found no significant difference between storage time or burial depth on viability and germination of several *Banksia* species in Australia; most seed viability was lost after 1 month at all burial depths in the 3 m topsoil stockpile. Hall et al. [30] found no significant effects of stockpiling topsoil from surface mines in Appalachia for 4 grass species over 8 months; however, seeds were only buried at 30 cm depth.

Ours is the only experiment that studied effects of stockpile size and burial depths > 3 m on seed viability and germination while concurrently measuring soil physical and chemical changes. Seed loss from stockpiling have mostly been attributed to in situ germination, predation, physical and mechanical damage or seed decomposition [14, 12, 29]. No studies provided direct evidence of causes for loss of seed viability. Rockich et al. [12] attributed seed viability loss of *Banksia* species in topsoil stockpiles to high temperature and water content. Riveria et al. [29] attributed loss of seed viability to in situ germination. Dickie et al. [14] suggested anaerobic conditions deep in stockpiles would be a factor in seed viability reduction. Our research showed seed viability declined in both aerobic and anaerobic conditions.

Until detailed studies determine how environmental factors in stockpiles regulate seed viability of boreal species at the cellular level, exact mechanisms for killing seeds remains uncertain. It appears likely that rapid loss of seed viability in small stockpiles and the upper 1 m of large stockpiles is mainly due to in situ germination, seed decay or rotting. Seed viability was negatively correlated with oxygen (4 species, r = -0.374 to -0.541, df = 30, P<0.05) suggesting aerobic processes led to mechanisms reducing seed viability. Seed viability of species was significantly (P <0.05, df = 30), positively correlated with methane (1 species, r = 0.479), ethylene (1 species, r = 0.479) and carbon dioxide (8 species, r = 0.366 to 0.546) and negatively correlated with available ammonium (21 species, r = -0.516 to -0.817), iron (3 species, r = -0.377 to -0.433), manganese (3 species, r = -0.381 to -0.401) and carbon dioxide (8 species, r = -0.359 to -0.561). The complexity of interactions among soil temperature, atmosphere and chemical environment makes it difficult to determine factors promoting in situ seed germination given the lack of knowledge on germination requirements for most species used in this research. The literature suggests increased soil temperature, carbon dioxide, methane and nitrate concentrations can enhance seed germination, but too great an increase can prevent germination [31]. Accelerated seed decay or rotting is attributed to enhanced microbial activity from stockpiling soil rich in organic carbon. Bacteria and fungi are considered a major cause of buried seed death, although few field studies addressed this hypothesis [31]. Seeds in warm, damp conditions lose viability sooner than those in cool, damp conditions [32]. Lower soil temperatures in winter constructed stockpiles would thus result in less seed death.

In large stockpiles, below 1 m, seed viability is likely lost due to mechanisms causing accelerated seed aging at the cellular level, caused by mechanisms from anaerobic conditions. Seed viability of many species was significantly (P <0.05), df = 30), positively correlated with oxygen (16 species, r = 0.387 to 0.621) and negatively correlated with available ammonium (9 species, r = -0.373 to -0.437), iron (3 species, r = -0.366 to -0.444), manganese (10 species, r = -0.362 to -0.543) methane (12 species, r = -0.362 to -0.558), ethylene (1 species, r = -0.356) and carbon dioxide (10 species, r = -0.359 to -0.561). Major environmental factors recognized as deleterious for seed survival are temperature, water content and oxygen [33, 34, 35]. Seeds of agricultural species in environments with reduced oxygen often retain viability longer than if stored with ample oxygen [32]. However, when seed water content is above a critical point (15% for barley) anaerobic environments are deleterious to their survival [34]. Seeds extracted from great depths in our large stockpiles had no signs of radical emergence although they were imbibed with water. Ibrahim et al. [34] found seeds of various agronomic species stored at water contents 15% or higher had increased subcellular damage and under anaerobic conditions only survived for short periods because no oxygen was available for subcellular repair. It is not surprising, given environmental conditions in large stockpiles, that seed viability was positively correlated with oxygen. Other causes of reduced seed viability include viruses, bacteria or toxic concentrations of compounds such as ethanol.

### Species response to stockpiling

*Geranium bicknellii* and *Dracocephalum parviflorum* resisted deleterious effects of stockpiling. Both are annual or biennial, early successional in boreal forest and seed banking [36, 37]. Their germination is promoted by heat from forest fires [38]. They were likely less affected by stockpiling because they have hard, impermeable seed coats [39, 40]. Dickie et al. [14] found only one species, *Juncus bufonius*, had numerous viable seeds at greatest burial depths in topsoil stockpiles in Derbyshire. They did not mention mechanisms leading to greater survival. Rivera et al. [29] found large seed mortality was negatively related to seed mass. Although we did not measure seed mass, small, light weight seed lost viability just as easily as large, heavy seeds.

Other species with a less dramatic decline in seed viability had harder seed coats than seeds of species that declined abruptly. Species with hard seed coats, (*Geranium bicknellii*, *Dracephalum parviflorum*, *Virburnum edule*, *Cornus canadensis*, *Vicia americana*, *Prunus pensylvanica*) generally maintained greater viability over time at multiple burial depths than seeds without hard seed coats (*Bromus ciliatus*, *Fragaria virginiana*, *Ribes hudsonianum*, *Vaccinium myrtilloides*). Seeds of *Vicia americana* that were not fully ripe and became soft after a short soaking lost more viability then seeds that were fully ripe.

Seeds with a hard coat that allows water exchange, like *Prunus pensylvanica*, lost viability quickly. Seeds with physical dormancy would not be as affected initially by stockpiling, unless the seed coat was scarified and made permeable. Seeds with physical dormancy that are impermeable to water are able to remain inactive and sustain low respiration rates. Rockich et al. [12] and Brophy [41] noted legume species with hard seed coats retained high viability in stockpiled topsoil.

Seeds that do not have physical dormancy are completely vulnerable to the surrounding deleterious environment. Species with low respiration rates or with persistent seed banks might tolerate greater burial depths in stockpiles; however, this was not the case for all species with persistent seed banks. *Rubus idaeus* and *Prunus pensylvancia* are shrubs with known seed banking capabilities and seeds can stay viable in the soil for 100 to 200 years [42, 43]. Loss of up to 100% viability at depths > 1 m in the stockpiles for these species is surprising considering their natural longevity in the soil. Once seed of most dry land species has imbibed water, respiratory activity and oxygen consumption increases [44]. Without oxygen at great depths in large stockpiles, seeds cannot repair cellular damage from initial influx of water into cells. Seeds in environments that facilitate respiration will lose viability more rapidly than those that do not, unless those species have mechanisms to keep respiration rates low (wetland species tolerant to anoxic conditions) or prevent the surrounding environment from influencing seed integrity, such as physical dormancy seeds.

### Stockpile construction methods

Direct placement of forest topsoil should be the preferred soil handling technique; however, stockpiling soil is a necessary component of reclamation and soils handling for any mine. There is little direction on how to construct stockpiles to maintain seed viability, and to a lesser extent, reduce negative effects on soil quality. Our research has shown that regardless of stockpile size most species will lose seed viability within a relatively short period of time (< 8 months) at depths below the surface of the stockpile; however, there are less detrimental changes to soil chemistry in small stockpiles. This research suggests the best solution to maintain a native seed bank and minimize nutrient losses from stockpiled boreal forest topsoil for future use in revegetation would be to construct several small stockpiles rather than fewer large stockpiles. This would increase the overall surface area of stockpiled soil, reducing the volume of anaerobic soil conditions. Rivera et al. [29] proposed construction of large stockpiles only for a short time to reduce seed loss in topsoil; however, our research suggests large stockpiles reduce seed viability more quickly than in small stockpiles.

Seed viability for a wide range of species could be maintained over the long term by building a new seed bank from newly established plants emerging from the former seed bank at the stockpile surface. However, space is often limited in the oil sands, preventing construction of several small stockpiles. Alternative placement locations such as former stockpiles constructed from reclamation materials other than upland topsoil is a potential solution to finding more available space.

Constructing stockpiles in winter or when soils are dry could reduce deleterious effects of stockpiling, at least in the short term. Anderson et al. [9] found wet constructed stockpiles became more anaerobic than dry constructed ones. In the boreal forest waiting until conditions are dry is not practical. Low temperatures can be used advantageously for constructing stockpiles to maintain seed viability and reduce impacts to soil quality, even though benefits will be short lived unless stockpiles are constructed to prevent topsoil from thawing and becoming saturated with water from snow melt. Capping topsoil that has been salvaged and stockpiled during frozen months with a thick layer of peat could help minimize thaw rate.

The long term effects of storing soil in the boreal forest on quality and quantity of organic carbon and effects on chemical properties needs to be determined. Research has shown stockpiling soil results in reduced aggregate stability making soils more prone to erosion and susceptible to compaction once replaced [45]. Stockpiling topsoil causes significant alterations to microbial community composition and abundance [46]. This research has demonstrated that various soil nutrients such as ammonium, potassium, calcium, manganese and iron become much more concentrated in their soluble forms after stockpiling. Specific revegetation methods and management may be required for reclaiming stockpiled topsoil to sustainable boreal forests due to the drastic changes of the soils chemical and physical state.

## Conclusions

Stockpiling boreal forest topsoil is deleterious to seeds and changes many soil chemical and soil atmosphere parameters. The effect is greatest with large stockpiles, fine texture soils and construction under non frozen conditions. Boreal forest topsoil is rapidly altered with storage. In both large and small stockpiles, seeds at depths below 1 m rapidly lost viability. Anaerobic soil conditions occurred rapidly and persisted at depths below 1 m in large stockpiles; in small stockpiles anaerobic conditions occurred, then became aerobic when monthly air temperatures fell below 0°C. In small stockpiles seed viability was lost because of aerobic that enhanced in situ germination and seed decay. Lack of oxygen combined with temperatures above 0°C and soil with enough water to imbibe seeds caused the rapid loss in seed viability in large stockpiles. Only seeds of *Geranium bicknellii* and *Dracocephalum parviflorum* had a high survival rate in stockpiles; both species have hard seed coats and are physically dormant.

Direct placement of boreal forest topsoil is the preferred soil handling technique; however, when stockpiling is required construction of more, small stockpiles would help retain seed and propagule viability in the short term and a greater surface area with viable seed and propagule banks would be created long term.
